# Social support detection from social media texts

**DOI:** 10.1371/journal.pone.0337476

**Published:** 2026-03-25

**Authors:** Zahra Ahani, Moein Shahiki Tash, Fazlourrahman Balouchzahi, Luis Ramos, Grigori Sidorov, Alexander Gelbukh, Rau´l Monroy

**Affiliations:** 1 Instituto Politécnico Nacional (IPN), Centro de Investigación en Computación (CIC), Mexico City, Mexico; 2 Tecnologico de Monterrey, Escuela de Ingenieria y Ciencias, Mexico City, Mexico; Educational Testing Service: ETS, UNITED STATES OF AMERICA

## Abstract

Social support, conveyed through a multitude of interactions and platforms such as social media, plays a pivotal role in fostering a sense of belonging, aiding resilience in the face of challenges, and enhancing overall well-being. This paper introduces Social Support Detection (SSD) as a Natural Language Processing (NLP) task aimed at identifying supportive interactions within online communities. We define SSD through three subtasks: (1) binary classification of whether a comment expresses social support or not social support, (2) binary classification of the intended support target (individual or group), and (3) multiclass classification of the specific group being supported, including Nation, Other, LGBTQ, Black Community, Religion, and Women. We conducted experiments on a manually annotated dataset of 9,998 YouTube comments. Traditional machine learning models were employed using various combinations of linguistic, psycholinguistic, emotional, and sentiment-based features. Additionally, neural network-based models incorporating word embeddings were evaluated to enhance performance across the subtasks. The results indicate a prevalence of group-oriented support in online discourse, highlighting broader societal dynamics. The findings show that integrating psycholinguistic and affective features with unigram representations improves classification performance. The best macro F1-scores achieved across the subtasks range from 0.72 to 0.82.

## Introduction

Social support is the provision of behaviors, communication, and interactions that convey care and value to individuals, fostering a sense of belonging and aiding in coping with life’s challenges [1]. Social support manifests in diverse ways, ranging from expressions of care and encouragement to practical assistance or guidance. Recognizing the presence of supportive individuals who offer various forms of aid can serve as a buffer against stress and safeguard both emotional and physical health. The support patients receive from shared content plays a crucial role in enhancing their self-care practices and overall health results [2]. Specifically, individuals coping with chronic illnesses, disabilities, or cancer often find social media platforms invaluable, as they offer opportunities to connect with peers or professionals for guidance in managing their long-term conditions effectively [3].

Although previous research on hope speech detection provides useful methodologies, hope speech and social support are distinct concepts. Hope speech generally refers to speech that promotes optimism and counters hate speech [4], while social support encompasses broader communicative behaviors, including empathy, encouragement, guidance, and tangible assistance.

In this context, hope speech is not treated as a subtype of social support. Instead, both are considered complementary forms of prosocial communication, with different scopes and objectives. While hope speech often directly responds to hateful content, social support emphasizes providing comfort and help to those in need.

In recent years, a heightened awareness of the detrimental impacts of hate speech, abusive language, and misogyny on social media platforms has led to a surge in research efforts focused on their detection through Natural Language Processing (NLP) [5].

While social media platforms offer users the freedom and anonymity to express their opinions and engage in instant feedback, this liberty also fosters an environment where individuals may exploit the platform to propagate discriminatory or harmful views targeting specific demographics [6]. Consequently, developing tools and techniques for detecting and mitigating such content has become imperative in creating safer digital environments and promoting respectful online discourse.

However, some argue that this approach can infringe on users’ freedom of expression [6,7]. Instead of solely focusing on identifying and removing negative content, an alternative strategy could involve promoting positive interactions and supporting content that contributes to social good. By encouraging and amplifying constructive and respectful communication, social media platforms can foster a more positive online environment while still respecting users’ rights to freely express their opinions. This dual approach not only mitigates the spread of harmful content but also actively contributes to a more supportive and inclusive digital community [8].

Despite the importance of promoting positive and supportive content, not many tasks have been done in this research area. In response to these challenges, our proposed approach offers an alternative but under-explored strategy to combat the negative atmosphere on social media platforms by promoting social support comments. Rather than solely focusing on identifying and filtering out negative content, our approach seeks to cultivate a more positive and supportive online environment by encouraging users to provide emotional comfort, encouragement, and advice to those facing challenges.

Online social support encompasses the assistance and emotional comfort offered via digital platforms such as social media, forums, and messaging apps. This type of support is crucial for individuals and groups facing various challenges, such as victims of wars or individuals from historically marginalized communities who may experience social, economic, or political disadvantages. Through these digital channels, individuals can connect with others who share similar experiences, access valuable resources, and receive empathy and encouragement. The anonymity and accessibility of online support networks often make them a vital lifeline for those who might not have access to traditional forms of support. Additionally, these platforms can provide real-time assistance, foster a sense of community, and help reduce feelings of isolation and loneliness. A detailed definition and social support are presented in section.

Previous research has shown that users in online communities both seek and provide support while occupying emergent social roles, such as support seekers or providers.

These roles arise through interaction, and early behaviors can influence long-term engagement. Understanding these dynamics helps analyze how social support is exchanged and received in online environments [9,10].

This work contributes to the understanding of supportive communication in online environments, with potential implications for reducing stress, enhancing coping mechanisms, and fostering inclusivity in digital communities.

Inspired by tasks in hate speech detection [11,12], an opposite task, social support detection (SSD) from text, is proposed. This task is modeled as a three-step classification process: (1) identifying whether a comment expresses support or not, (2) categorizing the type of support (e.g., emotional, informational), and (3) detecting the target group or individual receiving the support.

Data was collected from YouTube by first identifying videos containing supportive content. After preprocessing, a final random subset of 9,998 comments was selected for manual annotation. This resulted in 2,236 supportive comments and 7,762 non-supportive comments. Following data preparation, experiments were conducted using traditional machine learning models, including Logistic Regression (LR), Support Vector Machine with radial basis function kernel (SVM(rbf)), Support Vector Machine with a linear kernel (SVM (linear)), Decision Trees (DT) and Random Forest Classifier (RFC). Three different feature sets were used: (1) LIWC + emotion + sentiment features — where LIWC (Linguistic Inquiry and Word Count) is a psycholinguistic tool that analyzes text by counting words in various psychologically relevant categories, combined with additional emotion and sentiment indicators; (2) TF-IDF-based unigrams — frequency-based vector representation of words; and (3) a combination of LIWC, emotion, sentiment features, and TF-IDF unigrams. Results showed that combining psycholinguistic, emotional, and sentiment features with TF-IDF yielded the best performance, achieving F1-macro scores of 0.78 and 0.80 in Tasks 1 and 2, respectively. Additionally, different word embeddings (GloVe and FastText) and model architectures (CNN and BiLSTM) were used to generate predictions and compare them with traditional models.

The main contributions of this study are listed below:

- Study of social support for social good as a novel task in NLP,
- Developing annotation guidelines and generating the first specific social support detection dataset in English,
- Study of psycholinguistic features of social support for different levels: group, individual, and target groups,
- Providing benchmark experiments using traditional machine learning models and linguistic and psycholinguistic features.

## Definitions

Albrecht et al. [13] define social support as verbal and nonverbal communication between recipients and providers that reduces uncertainty about the situation, the self, the other, or the relationship, and functions to enhance the perception of personal control in one’s experience.

Although social support is helpful during stressful situations, [14] pointed out that the exchange of support does not only manifest during the crisis but is also an everyday occurrence in personal relationships.

The current research aligns with [14] definition of social support and views the exchange of comments and feedback between users and audiences as a form of social support occurring within their communication. Social support refers to”information leading the subject to believe that he is cared for and loved, esteemed, and a member of a network of mutual obligations” [15]. It is formed by the exchange of resources (i.e., verbal and nonverbal messages) between two or more individuals [16].

Studies have demonstrated that social support offers advantages to patients, such as dealing with challenging life circumstances [17], enhancing compliance with recommended treatment plans [18], and fostering better mental health [19]. In the management of chronic illnesses, social support plays a critical role in encouraging healthy behaviors and attaining favorable health results for patients. For instance, [18]. discovered that social support enhances individuals’ quality of life and diminishes psychological distress among those facing severe mental health issues.

Hence, social support is defined as”the emotional, informational, or practical assistance offered by others, including peers or community members”. This aid can be extended to individuals or groups, such as women, religious communities, or racial minorities like black community, aiding them in navigating challenges, enhancing their overall well-being, and fostering resilience.

## Related work

Despite the critical importance of promoting positive and supportive content, research in this area remains relatively sparse and often serves as a counterpoint to hate speech. While there is no directly comparable work focused solely on positive content in NLP, related research, such as hope speech [20] offers some insights. Recent studies on hate speech have effectively used psycholinguistic features—particularly those from LIWC—to enhance detection. For instance, ElSherief et al. [21] and Silva et al. [22] demonstrated that LIWC-based emotional and cognitive cues improve classification performance. Mathew et al. [8] used LIWC to analyze counter speech (non-hateful replies to hate speech meant to refute or counter it), revealing its linguistic contrasts with hate speech. Building on this foundation, our work applies similar features to the underexplored domain of supportive communication, offering a complementary perspective.

The phenomenon of hate speech and violent communication online is commonly referred to as cyberhate [23]. It involves the use of electronic communication technologies to propagate discriminatory or extremist messages, targeting not only individuals but also entire communities [24]. Hate speech encompasses various linguistic styles and actions, including insults, provocation, and aggression [25]. It can be categorized into different types, such as gendered hate speech, which targets specific genders or promotes misogyny, religious hate speech, which discriminates against various religious groups, and racist hate speech, which involves racial discrimination and prejudice against particular ethnicities or regions [26].

The feasibility of utilizing domain-specific word embeddings as features and a bidirectional LSTM-based deep model as a classifier for the automatic detection of hate speech was studied by [27]. Three datasets were used, with a total collection comprising 21,514 non-hate and 27,085 hate instances. To ensure balanced data, 16,260 instances for each label were used. This approach facilitated the detection of coded language by appropriately ascribing negative connotations to words. Additionally, the applicability of the transformer-based transfer learning language model (BERT) to the hate speech classification task was investigated, given its high-performance results across various NLP tasks. Experimental findings indicated that the combination of domain-specific word embeddings with the bidirectional LSTM-based deep model achieved an F1 score of 93%, while BERT achieved an F1 score of 96% when applied to a combined balanced dataset sourced from existing hate speech datasets.

Balouchzahi et al. [7] introduced PolyHope, the first multiclass hope speech detection dataset in English. The dataset creation process involved the collection of approximately 100,000 English tweets, which were preprocessed to yield around 23,000 tweets. A random subset of 10,000 tweets was subsequently selected for annotation, resulting in final statistics of Hope = 4175 and Not-Hope = 4081 post-annotation. They further fine-grained the type of hope into General, Realistic, and Unrealistic hopes. To assess the dataset’s performance, various baseline models were evaluated using diverse learning approaches, including traditional machine learning, deep learning, and transformer-based methods. The top-performing models for each learning approach demonstrated the average macro F1 scores for both binary and multiclass classification tasks on the PolyHope dataset, with transformers achieving better results, scoring 0.85 for binary classification and 0.72 for multiclass classification.

Palakodety et al. [28] analyzed an unfolding international crisis using a substantial corpus of YouTube comments, consisting of 921,235 English comments posted by 392,460 users, drawn from a total of 2.04 million comments by 791,289 users across 2,890 videos. Three primary contributions were highlighted. Firstly, the effectiveness of polyglot word embeddings in revealing precise language clusters was emphasized, leading to the development of a document language identification technique requiring minimal annotation. Its applicability and usefulness across various datasets involving multiple low-resource languages were showcased. Secondly, temporal trends in pro-peace and pro-war sentiment were examined, noting that during periods of heightened tension between the two nations, pro-peace sentiment in the corpus reached its peak. Lastly, in the context of politically charged discussions during a volatile situation, the study explored the potential of automatically identifying user-generated web content that might contribute to reducing hostile discourse. While practical applications remained limited, the task of hope-speech detection was introduced as a step toward better understanding such dynamics, with the best performance reported using n-grams (F1 score: 78.51%).

## Dataset development

### Data collection and processing

This research focuses on analyzing data collected from YouTube comments across 15 videos spanning various categories such as national identity, Black Community, women, religion, LGBTQ + , and others. The videos were manually selected based on their topical relevance to themes likely to elicit social support or emotional discourse. These included socially or emotionally charged subjects such as the war between Israel and Palestine, events involving the Black Community, public reactions to Cristiano Ronaldo, LGBTQ+ issues, and women’s rights. The selection was guided by the prominence of these topics in public discourse, their potential to generate supportive interactions, and the availability of open, high-engagement comment sections. The inclusion of the Cristiano Ronaldo video was motivated by the significant public response following personal events in his life (e.g., family matters and mental health), which sparked an outpouring of supportive comments.

All comments were posted between April 12, 2016, and February 13, 2024. This range represents the oldest and newest timestamps in our dataset. The full list of selected video URLs is provided in S1 Appendix for transparency and reproducibility.

Initially, we amassed 66,272 comments. After filtering out duplicate and non-English entries (with duplicates defined as exact text matches across or within videos), the dataset was refined to 42,695 comments.

Since we lacked prior knowledge of how many comments were truly supportive, we implemented a keyword-based sampling strategy to enhance the likelihood of selecting supportive content. We then selected 5,000 comments containing predefined supportive keywords and an additional 5,000 comments randomly. The keywords included phrases such as “support,” “stay strong,” “I’m here to help,” “I believe in you,” “inspiring,” and related terms. These were chosen based on prior literature [29] and manual inspection for their semantic connection to support-related language. They were not intended to serve as definitive labels of support, but rather as heuristic tools to guide sampling. Importantly, these keywords were used to identify general expressions of support and were not designed to capture any specific category of social support, such as informational or emotional support, or support given versus sought.

The full list of supportive keywords used is provided in S2 Appendix.

### Annotator selection

For annotator selection, three annotators were involved: two males and one female. Two of them (one male and one female) hold master’s degrees in computer science and possess proficient English language skills, while the third annotator, one of the authors of this paper, is a Ph.D. student in natural language processing with advanced English proficiency and a comprehensive understanding of the annotation schema and task objectives. Although the first two annotators were not native English speakers, both had completed their graduate education in English and had prior experience with academic reading and writing. This background ensured that all annotators were capable of understanding the nuanced meaning of online comments written in English. Initially, each annotator was provided with 100 sample comments and detailed annotation guidelines. These samples were used to help them familiarize themselves with the task. Individual meetings and interviews were then conducted to address any confusion and ensure a thorough understanding of the annotation procedure.

The full annotation set consisted of 9,998 comments. Each item was independently annotated by all three annotators, resulting in three annotations per comment. The comments were randomly shuffled and split into five batches of 2,000 items each for ease of assignment. Each annotator received all five batches over time and annotated every comment, with a 20-day deadline per batch. This setup allowed full triple-annotation coverage while maintaining manageable workload and scheduling.

The total annotation process spanned approximately 100 days, with each annotator annotating all 9,998 comments over five consecutive 20-day rounds.

### Annotation guidelines

The SSD task was structured as a three-step classification process. First, supportive comments were identified. Next, it was determined whether these supportive comments were directed toward an individual, a group, or a community. Finally, if the supportive comment was identified as being directed toward a group, the specific group was further identified. The guidelines for this process are described below.

- **Subtask 1 – Binary social support detection:** In this subtask, a given text is classified as either supportive or non-supportive:
  – **Social Support (label = SS):** Supportive statements promote understanding, empathy, and positive action. Therefore, a supportive comment is a statement or message that offers support, encouragement, admiration, or assistance to individuals or groups that are encountering difficulties or have accomplished something noteworthy. These comments aim to provide emotional support, boost morale, or acknowledge the achievements of others.
  – **Not Social Support (label = NSS):** The text does not convey any form of support, admiration, or encouragement.
- **Subtask 2 – Individual vs. Group:** In this subtask, each pre-identified in Subtask 1 supportive comment is further classified as support for an individual or support for a group:
  – **Individual:** If the text expresses support for a specific person or individual (e.g., Cristiano Ronaldo, Trump), it is labeled as Support for Individual.
  – **Group:** If the text expresses support for a group of people, community, tribe, nation, etc. (e.g., Muslims, Real Madrid, Black nations, LGBTQ), it is labeled as Support for Group.
- **Subtask 3 – Multiclass SS for Groups:** In this subtask, we aim to identify which community or group of people are targeted for social support by classifying the group supportive comments identified in Subtask 2 into the following categories:
  – **Women:** The text expresses support for women and promotes women’s rights and feminism.
  – **Black community:** The text expresses support for the black community and promotes black community rights.
  – **LGBTQ:** The text expresses support for the LGBTQ community and promotes LGBTQ community rights.
  – **Religion:** The text expresses support for a religion and its rights.
  – **Other:** The text expresses support for a community other than those listed above.
  – **Nation:** The text expresses support for a Nation and its rights.

### Annotation procedure

**Fig 1:**
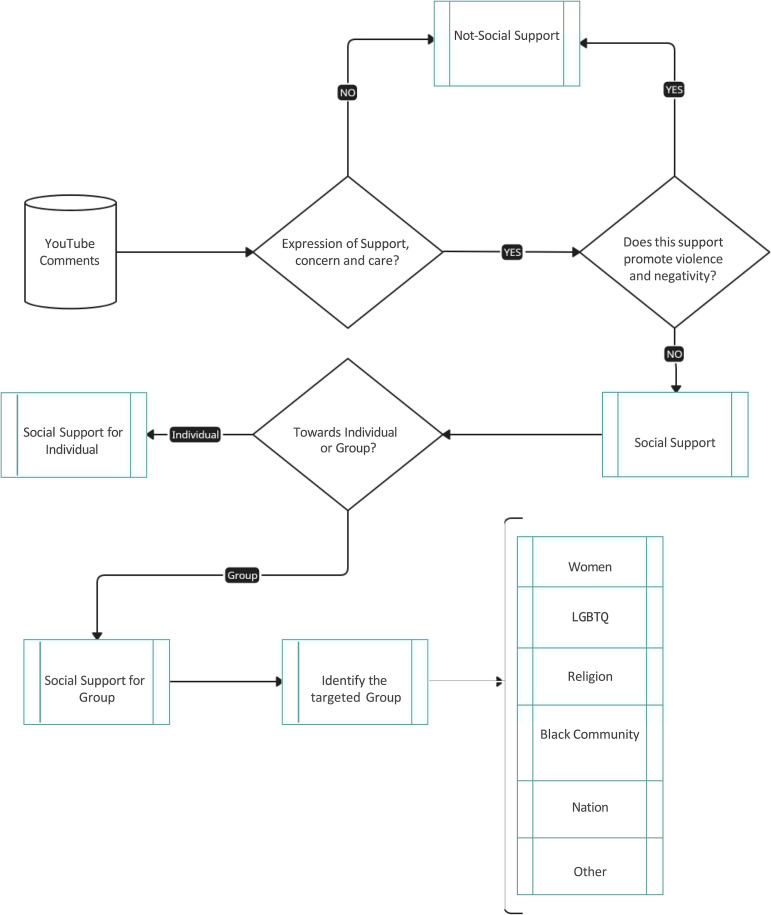
Overview of annotation procedure.

Detailed annotation guidelines and sample data were provided to the three chosen annotators to facilitate the creation of the proposed dataset. The annotators followed a structured process, as illustrated in Fig 1. Initially, they determined whether comments expressed support, concern, or care. As a substep of this first decision, annotators also assessed whether the identified support promoted violence or negativity; comments that did so were excluded from the dataset to ensure that only constructive and non-violent expressions of support were retained. If the comment was supportive and non-violent, annotators proceeded to a second level of analysis, distinguishing whether the support was directed towards an individual or a group. In cases where it pertained to a group, annotators further specified the group’s affiliation, such as Nation, Religion, Black Community, Women, LGBTQ, or Other. Conversely, if the comment did not exhibit support, annotators labelled it as Non-supportive. This process aimed to support comprehensive annotation and maintain dataset quality.

### Inter-annotator agreement

Inter-annotator agreement (IAA) measures the consistency among annotators while accounting for chance agreement. Fleiss’ Kappa coefficients, suitable for multiple annotators, were calculated for all tasks, resulting in scores of 0.711 for Task 1 (Binary Support), 0.899 for Task 2 (Individual vs Group), and 0.886 for Task 3 (Targeted Group), indicating a generally high level of reliability.

All 9,998 comments were independently annotated by three annotators for Task 1. For Task 2 and Task 3, annotation was performed on subsets following the hierarchical annotation design: Task 2 annotations were applied only to comments labeled supportive in Task 1, and Task 3 annotations only to comments labeled as ‘Group’ in Task 2. This naturally led to varying sample sizes per task.

Disagreements were resolved by majority voting per task. When two annotators agreed and one disagreed on a label, the majority label was chosen. Fleiss’ Kappa was calculated only on the subset of comments annotated by all three annotators for each task to ensure valid agreement measurement.

Distinguishing between individual and group support (Task 2) was challenging, particularly when comments lacked explicit target mentions or contained ambiguous support references. Annotators were instructed to select the most salient or explicit target in cases of mixed support. In ambiguous cases, majority voting determined the final label.

### Statistics of the dataset

Table 2 showcases the SSD dataset, which is organized into three hierarchical annotation levels with varying sample sizes. In Task 1, the number of Non-Social Support samples (7,762) greatly exceeds Social Support samples (2,236), indicating a higher prevalence of non-supportive comments. Task 2 shows more comments related to groups (1,813) than individuals (423); however, this distribution may reflect the specific nature of the selected videos rather than a broader tendency in user discussions. Task 3 reveals a wide distribution across specific categories, with Nation having the most samples (982) and Religion the fewest [20]. The higher number of comments about Nation can be attributed to the trending topic at the time of data collection, specifically the Israel-Palestine conflict. Other also has a significant number of samples (520) because it encompasses various additional categories within supportive comments.

**Table 1:** Examples of annotated comments.

Text	Task1	Task2	Task3
strong independent woman need help women powerful better smarter every ter men poor weak man protecting powerful woman	Supportive	Group	Women
need stop violence want bring peace black lives matter	Supportive	Group	Black Community
matter two boys love totally acceptable truly love fine people need understand	Supportive	Group	LGBTQ
god bless little heart may child every dream come true life ad well children survived nightmare may children civilians lost lives eternal peace red heart	Supportive	Group	Other
allah almighty give leaders muslim countries whic brave patriot nation stand islam	Supportive	Group	Religion
stand palestina insyaallah free one day	Supportive	Group	Nation
admired christiano ronaldo leadership good example team follows cant help comment adore	Supportive	Individual	—
incurred much losses trading think real market manipulated anyone help least tell wron	Non-Supportive	—	—

**Table 2:** Statistics of SSD dataset.

Tasks	Category	Number of samples
**Subtask1**	Social Support Not Social Support	2236 7762
**Subtask2**	Individual Group	423 1813
	Nation	982
	Other	520
**Subtask3**	LGBTQ Black Community	154 114
	Women	24
	Religion	19

The comments in the dataset vary widely in length, with a minimum of 3 characters, a maximum of 942 characters, and an average length of approximately 125 characters.

These differences likely stem from the inherent interest and sensitivity of the topics, the nature of the videos, and the sampling methods used. While this results in some class imbalance—particularly in Task 3—we retained these natural proportions to reflect the distribution of real-world discourse. To account for this imbalance during evaluation, we used macro-F1 scores, which weigh each class equally regardless of size. However, we acknowledge that some categories, especially those with very few instances (e.g., 24 or 19 cases), may not be large enough to support strong generalizations or robust statistical conclusions. Table 2 presents the statistics of the dataset. Table 1 further illustrates representative annotated examples from each task level to provide qualitative insight into the dataset.

## Feature analysis

This section delves into the complex interplay of psycholinguistic, emotional [30], and sentiment features utilized in our research. It not only outlines these features but also explores how they relate to various forms of social support. By understanding these dynamics, we gain a nuanced understanding of how language and emotions intersect with mechanisms of social support. Psycholinguistic attributes involve linguistic cues intertwined with psychological processes, extracted using LIWC software from social supportive comments. Emotional features [31] refer to the expression of specific emotions (e.g., joy, sadness, anger), along with their intensity and valence within communication, shedding light on the emotional dynamics of supportive interactions. Additionally, sentiment features reveal the overall sentiment conveyed in the text—whether positive, negative, or neutral—offering insights into the prevailing tone and attitude in supportive discourse.

### LIWC

The LIWC model has transformed psychological research by making language data analysis more robust, accessible, and scientifically rigorous. LIWC-22, the latest version, evaluates over 100 textual dimensions validated by esteemed research institutions worldwide. With over 20,000 scientific publications utilizing LIWC, it has become a widely recognized and trusted tool, enabling novel analytical approaches.

LIWC is a widely used computerized text analysis tool that enables researchers to examine the emotional, cognitive, structural, and process-related aspects of language. It works by performing frequency analysis of words in written text or speech [32]. The advantages of LIWC include its user-friendliness, ability to quantify complex psychological constructs, and strong empirical validation across various research fields. Recent studies have shown a growing trend in combining LIWC with machine learning techniques, especially for diagnosing mental disorders and investigating psychological traits. Such research often analyzes large volumes of text to identify relationships between everyday language use and characteristics like personality, social behavior, and cognitive patterns [32].

Despite these advantages, LIWC has limitations. One major issue is its reliance on predefined linguistic categories, which may not capture the nuances and variations of natural language [33,34]. Additionally, LIWC can struggle with accurately interpreting sarcasm, irony, and other subtle forms of language, potentially leading to misinterpretations.

In this study, we aim to employ machine learning and LIWC to detect social support.

We used a comprehensive set of LIWC-derived features covering multiple categories, including Summary Variables (overall language metrics such as word count), Linguistic Dimensions (e.g., pronouns, articles), and psychological dimensions such as Drives, Cognition, Affect, Social Processes, Culture, Lifestyle, Physical States, Motives, Perception, and aspects of Conversation. The average statistical relationships between these LIWC features and different categories of social support and non-supportive comments are detailed in Table 3.

**Table 3:** LIWC Feature Statistics for Social Support Analysis. All values (except Word Count) represent the average percentage of words in each category relative to total words per comment.

Feature	Social Support	Not Social Support	Group	Individual	Nation	Religion	Black Community	Women	Other	LGBTQ
**Word count**	44.0645	41.5249	44.0346	44.1927	43.9855	46.7228	45.1554	49.7326	42.6865	46.8500
**Function words**	11.7344	12.4165	11.5321	12.6014	10.9675	10.8692	11.8174	12.5538	11.8160	13.8851
**Drives**	3.0849	2.8181	3.0875	3.0736	3.1422	2.9367	2.8167	3.1643	3.3258	2.1412
**Cognition**	3.2936	4.1433	3.3058	3.2413	2.6595	3.2193	4.7268	3.6753	3.6126	5.2924
**Affect**	3.3550	2.1917	3.207	3.9894	3.3130	2.6355	1.7190	2.0784	3.4503	3.0571
**Social processes**	4.1968	3.6111	3.8995	5.4712	3.6828	3.3379	3.3794	4.7686	4.5102	3.5380
**Culture**	1.1013	0.9521	1.2687	0.3841	1.5139	4.4231	2.7719	0.3347	0.7258	0.1812
**Lifestyle**	1.6341	1.1859	1.5007	2.2061	1.6428	5.0315	0.5	0.6790	1.6762	0.4349
**Physical**	0.4253	0.3663	0.4414	0.3564	0.3394	0.1645	0.5611	0.2457	0.4902	0.9029
**States**	0.2726	0.3464	0.2803	0.2394	0.2436	0.1080	0.3325	0.5148	0.3219	0.3204
**Motives**	2.5244	2.1321	2.4793	2.7175	2.6755	1.8255	1.5546	2.2067	2.3508	2.4705
**Perception**	2.5254	2.8775	2.5355	2.4818	2.3648	1.9381	3.6379	2.8534	2.5619	2.7431
**Conversation**	0.4950	0.5146	0.4795	0.5616	0.5037	0.2757	0.4729	0.308	0.4431	0.5049

The values presented in Table 3 represent the **average percentages** of words in each LIWC category relative to the total word count per comment, except for “Word Count,” which indicates the average number of words per comment. All other categories, such as “Function Words,” reflect the **average percentage** of words belonging to that category within the comments.

## LIWC feature analysis

To aid interpretation, we briefly describe the main LIWC categories used in this analysis. LIWC (Linguistic Inquiry and Word Count) assigns words in texts to psychologically meaningful categories based on validated dictionaries. Each category reports the proportion of words in a text belonging to that dimension. The following are the relevant LIWC categories analyzed in this study:

- **Word Count (WC)**: Total number of words per comment.
- **Function Words**: Articles, pronouns, prepositions, conjunctions, auxiliary verbs, and other structural words.
- **Drives**: Motivational categories including achievement, affiliation, power, reward, and risk.
- **Cognition**: Words related to thinking processes such as insight, causation, certainty, and discrepancy.
- **Affect**: Emotion-related words, including positive and negative emotions, anxiety, anger, and sadness.
- **Social Processes**: Words referring to human interactions such as family, friends, and communication.
- **Culture**: Words related to politics, ethnicity, religion, and technology.
- **Lifestyle**: Terms about work, school, and home life.
- **Physical**: Health-related words including illness, wellness, mental health, and substances.
- **States**: Words referring to temporary internal conditions like tiredness or hunger.
- **Motives**: Words reflecting internal motivational states or goals.
- **Perception**: Words about seeing, hearing, feeling, movement, space, and attention.
- **Conversation**: Informal language features such as assent, fillers, disfluencies, and internet slang.

**Word Count (WC)** refers to the average number of words used per comment. In our dataset, supportive comments exhibit slightly higher word counts on average compared to non-supportive ones, indicating that supportive messages tend to be more elaborated.

**Function Words** in LIWC include pronouns, impersonal pronouns, articles, prepositions, auxiliary verbs, common adverbs, conjunctions, and negations. These words play a structural role in language and are often used to explore communication style and psychological state. In our dataset, the average proportion of function words is slightly lower in supportive comments than in non-supportive ones. Individual-targeted comments also show a lower proportion compared to group-targeted comments. Comments related to LGBTQ topics contain a relatively higher proportion of function words.

**Drives** in LIWC represent motivational dimensions such as affiliation, achievement, power, reward, and risk. Supportive comments show a slightly higher average proportion of Drive-related words compared to non-supportive ones. The difference between group- and individual-targeted comments is small, suggesting similar usage patterns across these categories. Within the group support subcategories, comments labeled as “Other” display a higher proportion of Drive-related language.

**Cognition** in LIWC includes words related to cognitive processes such as causation, insight, certainty, and discrepancy. In our dataset, non-supportive comments contain slightly higher percentages of cognition-related words than supportive ones. Group-targeted comments also show somewhat higher values than individual-targeted ones. Comments related to LGBTQ topics show elevated cognitive word use

**Affect** in LIWC captures emotion-related language, including both positive and negative emotions, as well as specific states like anxiety, anger, and sadness. Supportive comments show a higher average proportion of affective words than non-supportive ones. Individual-targeted comments also exhibit higher affective word use than group-targeted ones.

**Social Processes** covers words related to human interactions, such as family, friends, and communication. Supportive comments show a slightly higher average proportion of social process words than non-supportive comments. Individual comments tend to use more social process language compared to group comments.

**Culture** covers words related to politics, ethnicity, religion, and technology. Supportive comments tend to have a slightly higher proportion of cultural terms than non-supportive comments. Group discussions show higher usage of culture-related words than individual comments. The “religion” subgroup within group discussions shows a notably higher value.

**Lifestyle** includes words about work, school, home life, and employment. Supportive comments exhibit a higher average proportion of lifestyle-related words than non-supportive ones. Individual comments show slightly more engagement with lifestyle terms compared to group comments. The “religion” subgroup is elevated in this category.

**Physical** refers to health-related words including illness, wellness, mental health, and substances. Supportive comments show slightly higher usage of physical terms than non-supportive comments. Group comments tend to use more physical words than individual comments. The “religion” subgroup shows lower proportions.

**States** include words about temporary internal conditions such as tiredness or hunger. Non-supportive comments and group interactions show slightly higher average proportions of state-related words. The “Women” subgroup displays elevated values in this category.

**Motives** reflect internal motivational states or goals. Supportive comments, individual comments, and comments related to national identity show higher average proportions of motive-related words.

**Perception** includes words related to seeing, hearing, feeling, movement, space, and attention. Non-supportive comments, group comments, and those relating to Black individuals show higher average proportions of perception words.

**Conversation** includes informal language features such as assent, fillers, disfluencies, and internet slang. Non-supportive comments, individual comments, and LGBTQ-related comments exhibit higher average proportions of conversational markers.

All observations described above are descriptive and no statistical tests were performed to assess the significance of differences across comment categories.

### Emotions

This study employed the NRC Emotion Lexicon (version 1.0) [35] to analyze emotional content in comments associated with different types of social support. The NRC Emotion Lexicon is a manually curated resource that maps English words to eight basic emotions: anger, anticipation, disgust, fear, joy, sadness, surprise, and trust.

To compute emotion scores for each comment, we matched every word to the NRC Emotion Lexicon. For each emotion, we retrieved the corresponding emotion-intensity score for every matched word and summed these values across the comment. Then, for each support category (e.g., Social Support, Individual, Group, Nation, etc.), we calculated the average total emotion intensity per comment by averaging these summed scores across all comments within that class. Consequently, the resulting values in Table 4 represent average cumulative emotion intensities, which can exceed 1 because they reflect total emotion strength rather than normalized proportions by word count. These scores were then averaged across all comments within each support category.

**Table 4:** Emotion scores for social support subtasks.

Subtasks	Labels	Anger	Anticipation	Disgust	Fear	Joy	Sadness	Surprise	Trust
**Subtask1**	**Social Support**	0.6399	1.2464	0.5223	1.0840	1.6923	1.0035	0.4190	1.6265
	**Not Social Support**	0.6946	0.9738	0.4987	0.9263	1.0332	0.8737	0.4408	1.2711
**Subtask2**	**Individual**	0.4066	1.6548	0.4609	0.8723	2.1583	0.6595	0.4893	1.8983
	**Group**	0.6944	1.1511	0.5366	1.1334	1.5835	1.0838	0.4026	1.5631
**Subtask3**	**Nation**	0.5651	0.8788	0.4327	0.9501	1.2932	0.8156	0.3319	1.2219
	**Other**	0.6596	1.3807	0.5057	1.2442	1.7615	1.0730	0.4211	1.8307
	**LGBTQ**	0.9675	1.5519	0.9935	1.2922	2.7402	1.1818	0.6623	1.8701
	**Black Community**	1.4824	1.4736	0.8508	1.8508	1.4736	3.2894	0.4912	2.3947
	**Religion**	0.8947	2.6315	0.6315	1.6315	2.6842	0.9473	0.5263	3.6315
	**Women**	1.0833	2.0416	0.9583	1.4166	1.8333	1.2916	0.7083	2.1666

The emotion scores quantify the aggregate strength of words associated with each of the eight emotions within a comment, computed as the sum of lexicon-assigned emotion weights divided by the comment’s total word count (i.e., a weighted proportion); higher scores indicate greater presence or intensity of the respective emotion.

Table 4 shows the average emotion scores for different categories of comments, separated into supportive and non-supportive groups, as well as subgroups such as individual vs. group comments and demographic themes.

The data show that positive emotions such as *joy* and *trust* tend to have higher average scores in supportive comments compared to non-supportive ones across most categories. For example, joy is notably higher in individual and LGBTQ-related supportive comments. Negative emotions such as *anger* and *fear* display more nuanced patterns, with variations across categories but no clear overall trend. Emotional expression varies across demographic groups, reflecting the complex interplay of emotions and social dynamics in different contexts.

It is important to note that these emotion scores are descriptive and based on lexical matching; no statistical significance tests have been conducted to assess differences.

Therefore, these findings should be interpreted cautiously as preliminary insights into emotional patterns in supportive discourse.

### Sentiment analysis

The Social Support dataset was analyzed for sentiment using the VADER (Valence Aware Dictionary and sEntiment Reasoner) sentiment analysis tool [36]. VADER is a rule-based sentiment analysis software specifically tuned for social media text. It uses a combination of a sentiment lexicon and heuristics to assign polarity scores to a given piece of text. Each text receives a compound score ranging from −1 (extremely negative) to +1 (extremely positive), along with proportions for negative, neutral, and positive sentiment.

To assign sentiment labels, we used VADER’s compound score: texts with scores above 0.05 were labeled as positive, below −0.05 as negative, and those in between as neutral.

Table 5 showcases the proportion of texts exhibiting negative, neutral, or positive sentiment within different categories. Across most categories, neutral sentiment appears to be the most prevalent, followed by either positive or negative sentiment, though the balance between the two varies. Non-supportive contexts generally exhibit slightly higher proportions of negative sentiment compared to supportive contexts, where neutral sentiment tends to dominate. Individual and group contexts show a relatively balanced distribution between neutral and positive sentiments. There are also noticeable differences in sentiment distribution across demographic groups, with variations particularly evident among the Black community and women, where negative sentiment appears more pronounced.

**Table 5:** Sentiment analysis scores for social support subtasks.

Subtasks	Labels	Negative	Neutral	Positive
**Subtask1**	**Social Support**	0.1498	0.4702	0.3798
	**Not Social Support**	0.1747	0.5741	0.2511
**Subtask2**	**Individual**	0.1183	0.4233	0.4583
	**Group**	0.1572	0.4812	0.3615
**Subtask3**	**Nation**	0.1360	0.4830	0.3809
	**Other**	0.1990	0.4497	0.3511
	**LGBTQ**	0.1171	0.4880	0.3947
	**Black Community**	0.2070	0.5780	0.2149
	**Religion**	0.1176	0.4826	0.3996
	**Women**	0.1714	0.5844	0.2441

Note: The numbers in Table 5 represent proportions (or percentages) of all statements for the relevant class.

These findings suggest that sentiment expression may be shaped by the social role of the speaker and demographic context. However, no statistical significance tests were conducted, so these observations should be interpreted as preliminary trends.

## Experiments

This section presents our experimental setup for detecting social support in online comments. We evaluate both traditional machine learning and deep learning models to classify supportive content, identify support types, and determine the target (individual or group). The experiments are based on a manually annotated YouTube dataset and aim to establish strong baselines using various feature sets, including psycholinguistic, emotional, sentiment, and TF-IDF-based representations. By comparing model performance across different inputs and architectures, we assess the effectiveness of linguistic and neural approaches for this novel NLP task.

### Traditional machine learning models

We utilize five traditional machine learning classifiers for detecting social support: Logistic Regression (LR), Support Vector Machine (SVM) with both radial basis function (RBF) and linear kernels, Decision Tree (DT), and Random Forest Classifier (RFC), all implemented using the scikit-learn library [37]. To enhance model robustness and potentially improve predictive performance, we explored ensemble methods based on both hard and soft voting strategies. Hard voting determines the final predicted class by majority vote, where each classifier casts one vote and the class with the most votes is selected. Soft voting, on the other hand, averages the predicted class probabilities from all classifiers and selects the class with the highest average probability, which can better capture classifier confidence. All five classifiers were included in the ensembles and combined using scikit-learn’s VotingClassifier with default parameters. The classifiers were trained using default hyperparameters to provide a fair baseline comparison rather than tuning individual models. The input features consist of psycholinguistic, emotional, and sentiment attributes extracted at the individual comment level, not aggregated across groups. Specifically, LIWC was used to compute numerical scores for each comment across categories such as function words, cognitive processes, affect, and social processes. Emotion features were obtained from the NRC Emotion Lexicon, which assigns scores for eight emotions (e.g., anger, joy, trust) based on detected emotion words in each comment. Sentiment features were generated using the VADER tool, providing negative, neutral, and positive sentiment probabilities per comment. These features form the input vectors for the classifiers. The aggregated feature statistics presented in Tables 3–5 serve only for descriptive analysis and are not directly used in model training. Additionally, pandas was used for data handling, scipy for sparse matrix operations, nltk for text preprocessing, and numpy for numerical computations throughout the experiments.

### Preprocessing

Initially, the data preprocessing involved removing duplicate comments and selecting only English comments. For general text standardization, tokenization, lowercasing, punctuation removal, stopword elimination, and stemming or lemmatization were performed. Emojis and emoticons were converted into textual representations using the emotion library [35]. Abbreviations were expanded to their full forms using a predefined dictionary, and additional punctuation and stopwords were removed to refine the text.

However, different feature extraction tools required tailored preprocessing: for LIWC, VADER, and the NRC lexicon, stemming, lemmatization, and stopword removal were not applied, as these steps could reduce the accuracy of lexicon matching. Instead, analyses with these tools were performed on texts after lowercasing and minimal cleaning (e.g., emoji conversion), preserving word forms and stopwords. In contrast, TF-IDF and word embedding-based models utilized fully preprocessed texts, including stemming/lemmatization and stopword removal, to optimize vocabulary representation and reduce noise.

### Feature extraction

We extracted multiple types of features to comprehensively represent the textual content of each comment for classification.

Psycholinguistic features: Using the Linguistic Inquiry and Word Count (LIWC) tool [38], we obtained numerical scores for categories such as function words, cognitive processes, affective states, social processes, culture, lifestyle, motives, perception, and more. Each category quantifies the prevalence of psychologically relevant word classes within a comment.

Emotional features: The NRC Emotion Lexicon [35] provided eight emotion intensity scores per comment, covering anger, anticipation, disgust, fear, joy, sadness, surprise, and trust. These capture the emotional tone present in the text.

Sentiment features: VADER sentiment analysis [36] was used to compute normalized scores representing negative, neutral, and positive sentiment proportions for each comment. VADER is optimized for social media text and captures subtle sentiment nuances.

Lexical features: We extracted TF-IDF weighted word unigrams to capture the frequency and importance of lexical patterns in the dataset. These features were used as the primary textual representation for the machine learning classifiers.

### Model training and predictions

In all experiments, we utilized a 5-fold cross-validation approach for both training and evaluating the ML models. Evaluation and comparison were conducted based on the average weighted and macro scores across all folds. Comprehensive results are elaborated upon in the Results section.

### Deep learning

Two deep learning models, namely Convolutional Neural Network (CNN) and Bidirectional Long Short-Term Memory (BiLSTM), were trained separately using Global Vectors for Word Representation (GloVe) and FastText embeddings. The models were implemented using the Keras library with TensorFlow backend [39]. A Keras tokenizer was fitted on the dataset texts to convert all texts into sequences. The maximum sequence length was set to the length of the longest comment, and all sequences were padded to this length. Vectors were obtained from the word embedding matrix for each comment, after which the input sequences were created and fed to the deep learning models. The hyperparameters used for both models are detailed in Table 6. Notably, both models share identical parameter settings as they were optimized for fair baseline comparison. Each model was trained for 50 epochs per fold using 5-fold cross-validation.

**Table 6:** Parameters for deep learning models.

Parameters	CNN	BiLSTM
Epochs	50 per fold	50 per fold
Optimizer	Adam	Adam
Loss	categorical crossentropy	categorical crossentropy
Embedding size	300	300
Learning rate (lr)	0.001	0.001
Dropout	0.1	0.1
Activation	softmax	softmax

## Results

The machine learning models were evaluated across three distinct classification steps, as presented in Table 2. Importantly, each level of our experiment was augmented with different feature combinations, namely LIWC+Emotions and sentiment features only, TF-IDF only, and a combination of all features. This approach allowed us to systematically investigate the impact of different feature sets on the performance of various models across different classification tasks. Through this comprehensive analysis, we aimed to identify the most effective model-feature combinations for accurate and reliable social support detection. We conducted experiments using CNN and BiLSTM models with GloVe and FastText embeddings across three subtasks, and present the results in Table 10.

### Social support detection with LIWC, emotion, and sentiment features

Table 7 presents the classification results using psycholinguistic features extracted from LIWC, alongside emotional and sentiment-based features. These handcrafted features were used to train several traditional machine learning classifiers to evaluate their effectiveness across the three subtasks.

**Table 7:** Classification results using LIWC, emotions and sentiments features.

Models	Avg. weighted scores			Avg. macro scores			Accuracy
	Precision	Recall	F1-score	Precision	Recall	F1-score	
Subtask1							
**LR**	**0.8151**	**0.8286**	**0.8111**	**0.7737**	**0.6794**	**0.7061**	**0.8286**
**SVM(rbf)**	0.8137	0.8154	0.7733	0.8095	0.6083	0.6265	0.8154
**SVM (linear)**	0.8179	0.8297	0.8080	0.7873	0.6688	0.6977	0.8297
**DT**	0.7512	0.7487	0.7497	0.6402	0.6428	0.6412	0.7487
**RFC**	0.8229	0.8288	0.8000	0.8098	0.6492	0.6785	0.8288
**Soft voting**	0.8229	0.8388	0.8066	0.7866	0.6721	0.7033	0.8312
**Hard voting**	0.8267	0.8326	0.8064	0.8124	0.6597	0.6905	0.8326
	**Subtask2**						
**LR**	**0.8680**	**0.8756**	**0.8688**	**0.8130**	**0.7506**	**0.7751**	**0.8756**
**SVM(rbf)**	0.8443	0.8403	0.7952	0.8535	0.5887	0.6066	0.8403
**SVM (linear)**	0.8658	0.8729	0.8661	0.8095	0.7469	0.7709	0.8729
**DT**	0.8030	0.7996	0.8010	0.6759	0.6800	0.6774	0.7996
**RFC**	0.8646	0.8698	0.8521	0.8435	0.6940	0.7335	0.8698
**Soft voting**	0.8648	0.8729	0.8629	0.8191	0.7306	0.7613	0.8729
**Hard voting**	0.8688	0.8752	0.8620	0.8380	0.7195	0.7558	0.8752
	**Subtask3**						
**LR**	**0.7056**	**0.7010**	**0.7000**	**0.5666**	**0.5940**	**0.5666**	**0.7010**
**SVM(rbf)**	0.5807	0.6321	0.5801	0.3355	0.2974	0.2973	0.6321
**SVM (linear)**	0.6844	0.6784	0.6773	0.5269	0.5606	0.5303	0.6784
**DT**	0.6450	0.6348	0.6358	0.4757	0.4851	0.4703	0.6348
**RFC**	0.7149	0.7308	0.7031	0.5222	0.4302	0.4500	0.7308
**Soft Voting**	0.7297	0.7369	0.7241	0.6689	0.5082	0.5490	0.7369
**Hard voting**	0.7167	0.7275	0.7082	0.6221	0.5011	0.5234	0.7275

In Subtask 1, the Logistic Regression (LR) model achieved the highest macro

F1-score of 0.7061, slightly outperforming other models such as SVM with linear and RBF kernels, Decision Tree (DT), and Random Forest Classifier (RFC). While ensemble models like Soft Voting and Hard Voting showed competitive performance, the margin of improvement was minimal. This trend was also observed in Subtask 2, where LR again obtained the highest macro F1-score of 0.7751. In Subtask 3, although overall performance decreased due to the increased complexity of the task, LR remained the top-performing model with a macro F1-score of 0.5666.

Interestingly, in all three subtasks, the performance differences between LR and other models such as SVM and RFC were relatively small, suggesting that the selected features offer a comparable level of informativeness across models. This indicates that Logistic Regression, a relatively simple yet interpretable model, is a strong candidate for social support classification when relying on LIWC, emotion, and sentiment features.

### Social support detection using TF-IDF word unigrams

In this section, we report the results of our experiment using TF-IDF weighted word unigram features. Unigrams refer to individual tokens (words) extracted from the raw text, without applying stemming or lemmatization. We used the TfidfVectorizer from the scikit-learn library to convert the text into a numerical feature space, where each feature represents the Term Frequency–Inverse Document Frequency (TF-IDF) score of a unigram in the document. The TF-IDF values were computed based solely on the training data to avoid information leakage.

Table 8 presents the model performance across different classification tasks. For subtask 1, the Soft Voting ensemble achieved the highest overall performance, outperforming individual classifiers. A similar trend was observed in subtasks 2 and 3, where Soft Voting continued to yield superior results. This ensemble method combines predictions from multiple base classifiers using their predicted probabilities rather than hard labels, effectively balancing diverse decision boundaries and improving generalization.

**Table 8:** Classification results using Unigrams with TF-IDF values.

Models	Avg. weighted scores			Avg. macro scores			Accuracy
	Precision	Recall	F1-score	Precision	Recall	F1-score	
Subtask1							
**LR**	0.8547	0.8608	0.8491	0.8329	0.7364	0.7682	0.8608
**SVM(rbf)**	0.8530	0.8591	0.8466	0.8324	0.7311	0.7636	0.8591
**SVM (linear)**	0.8516	0.8589	0.8493	0.8207	0.7432	0.7707	0.8589
**DT**	0.8063	0.8092	0.8076	0.7246	0.7172	0.7206	0.8092
**RFC**	0.8463	0.8536	0.8405	0.8213	0.7227	0.7539	0.8536
**Soft voting**	**0.8543**	**0.8611**	**0.8512**	**0.8265**	**0.7447**	**0.7733**	**0.8611**
**Hard voting**	0.8541	0.8602	0.8482	0.8326	0.7348	0.7667	0.8602
	**Subtask2**						
**LR**	0.8604	0.8622	0.8362	0.8554	0.6589	0.6977	0.8622
**SVM(rbf)**	0.8647	0.8698	0.8503	0.8490	0.6877	0.7286	0.8698
**SVM (linear)**	0.8632	0.8716	0.8615	0.8184	0.7281	0.7594	0.8716
**DT**	0.8346	0.8376	0.8358	0.7354	0.7243	0.7292	0.8376
**RFC**	0.8653	0.8716	0.8559	0.8412	0.7037	0.7426	0.8716
**Soft voting**	**0.8704**	**0.8779**	**0.8689**	**0.8279**	**0.7423**	**0.7730**	**0.8779**
**Hard voting**	0.8641	0.8707	0.8537	0.8410	0.6983	0.7376	0.8707
	**Subtask3**						
**LR**	0.7848	0.7909	0.7816	0.6453	0.5192	0.5486	0.7909
**SVM(rbf)**	0.7837	0.7915	0.7824	0.6467	0.5454	0.5708	0.7915
**SVM (linear)**	0.7995	0.8064	0.8010	0.6936	0.6275	0.6458	0.8064
**DT**	0.7929	0.7876	0.7873	0.7105	0.6864	0.6807	0.7876
**RFC**	0.8249	0.8290	0.8227	0.7245	0.6522	0.6667	0.8290
**Soft voting**	**0.8280**	**0.8273**	**0.8239**	**0.8074**	**0.7043**	**0.7262**	**0.8273**
**Hard voting**	0.8051	0.8102	0.8033	0.7202	0.6046	0.6306	0.8102

Notably, Logistic Regression and linear SVM models also demonstrated strong individual performance. However, their combination through Soft Voting produced more balanced and stable results across all evaluation metrics. These findings indicate that TF-IDF word unigrams alone provide a strong and reliable signal for social support classification in English-language social media comments, highlighting the effectiveness of simple lexical features in capturing supportive communication patterns.

### Social support detection with the combination of all features

In this section, we integrated a combination of all features including LIWC, emotions, sentiment scores, and Unigram with TF-IDF values. Table 9 presents the outcomes of our experiments across different classification tasks. In the first subtask, SVM (Linear) emerges as the top-performing model, surpassing others in terms of performance metrics. Moving to the second subtask, soft voting stands out with the highest performance value among the models considered. Transitioning to the third subtask, hard voting demonstrates superior results compared to other models. These findings highlight the varied strengths of different models when leveraging a comprehensive feature set, underscoring the importance of selecting appropriate models tailored to the specific task requirements.

**Table 9:** Classification results using all features.

Models	Avg. weighted scores			Avg. macro scores			Accuracy
	Precision	Recall	F1-score	Precision	Recall	F1-score	
Subtask1							
**LR**	0.8175	0.8306	0.8145	0.7753	0.6862	0.7127	0.8306
**SVM(rbf)**	0.8115	0.8134	0.7696	0.8072	0.6031	0.6194	0.8134
**SVM (linear)**	**0.8558**	**0.8626**	**0.8557**	**0.8190**	**0.7602**	**0.7830**	**0.8626**
**DT**	0.7889	0.7905	0.7897	0.6974	0.6946	0.6959	0.7905
**RFC**	0.8465	0.8492	0.8292	0.8391	0.6944	0.7303	0.8492
**Soft voting**	0.8500	0.8565	0.8433	0.8285	0.7256	0.7579	0.8565
**Hard voting**	0.8534	0.8576	0.8424	0.8407	0.7189	0.7544	0.8576
	**Subtask2**						
**LR**	0.8678	0.8752	0.8684	0.8126	0.7505	0.7747	0.8752
**SVM(rbf)**	0.8497	0.8398	0.7924	0.8699	0.5841	0.5997	0.8398
**SVM (linear)**	**0.8783**	**0.8828**	**0.8792**	**0.8186**	**0.7808**	**0.7969**	**0.8828**
**DT**	0.8416	0.8465	0.8437	0.7498	0.7316	0.7399	0.8465
**RFC**	0.8709	0.8698	0.8472	0.8731	0.6774	0.7204	0.8698
**Soft voting**	0.8828	0.8886	0.8798	0.8528	0.7561	0.7910	0.8886
**Hard voting**	0.8791	0.8832	0.8702	0.8614	0.7285	0.7696	0.8832
	**Subtask3**						
**LR**	0.7116	0.7120	0.7078	0.6126	0.5720	0.5717	0.7120
**SVM(rbf)**	0.5837	0.6310	0.5740	0.3476	0.2948	0.2927	0.6310
**SVM (linear)**	0.7260	0.7186	0.7193	0.5466	0.5746	0.5482	0.7186
**DT**	0.7698	0.7677	0.7670	0.6465	0.6448	0.6348	0.7677
**RFC**	0.7620	0.7793	0.7590	0.5392	0.5024	0.5145	0.7793
**Soft Voting**	0.7736	0.7848	0.7703	0.6375	0.5711	0.5877	0.7848
**Hard voting**	**0.7980**	**0.8041**	**0.7959**	**0.7248**	**0.5998**	**0.6355**	**0.8041**

The integration of all feature types provided a richer and more discriminative representation of the input text, allowing models to better capture the multifaceted nature of social support. Notably, while linear SVM excelled in Subtasks 1 and 2 due to its ability to effectively separate high-dimensional data, the ensemble-based approaches (Soft and Hard Voting) outperformed in Subtask 3, which is inherently more challenging. This suggests that combining the outputs of diverse classifiers helps mitigate individual model biases and enhances robustness, especially in complex multi-class scenarios.

Overall, the use of hybrid feature sets and ensemble learning significantly boosts performance across tasks, reflecting the complementary contributions of linguistic, affective, and statistical text representations.

In Table 9, the comparison across three levels of SSD reveals the performance of various models with different feature combinations. In the first subtask, the combination of LIWC, Emotions and sentiments features, and Unigram with TF-IDF values with the SVM (linear) model demonstrate superior performance. Moving to the second subtask, we observe that the combination of LIWC, Emotions and sentiment analysis, and Unigram with TF-IDF values with the soft voting model yields the highest value. Finally, in the third subtask, the TF-IDF feature with the soft voting model exhibits the highest performance. These findings underscore the influence of both feature set and model choice on the effectiveness of SSD across different subtasks.

### Deep learning

Table 10 provides an overview of model performance across various tasks, with a focus on the macro F1-score, which is considered a robust metric for evaluating model performance in tasks with imbalanced classes. Across different word embeddings (GloVe and FastText) and model architectures (CNN and BiLSTM), the configurations yielding the highest macro F1-scores are of particular interest. For example, in Task 1, using Glove embeddings with the BiLSTM model resulted in a macro F1-score of 0.7611, with precision and recall scores of 0.7751 and 0.7587, respectively. These values indicate a reasonably balanced trade-off between the two metrics, justifying the reliability of the F1-score in this case. Similarly, in Task 2 and Task 3, certain configurations achieved macro F1-scores of 0.8184 and 0.7235 respectively, reflecting strong generalization across all classes, particularly when both precision and recall are consistently high.

**Table 10:** Data classification results for deep learning models.

Word Embedding	Tasks	Model	Weighted scores			Macro scores			Accuracy
			precision	recall	F1-score	precision	recall	F1-score	
**GloVe**	Task1	CNN	0.8406	0.8449	0.8355	0.7819	0.7520	0.7526	0.8449
		BiLSTM	**0.8392**	**0.8448**	**0.8389**	**0.7751**	**0.7587**	**0.7611**	**0.8448**
**FastText**		CNN	0.8007	0.8099	0.8009	0.7231	0.6984	0.7028	0.8099
		BiLSTM	0.8130	0.8122	0.8082	0.7280	0.7287	0.7204	0.8122
**GloVe**	Task2	CNN	**0.8913**	**0.8957**	**0.8921**	**0.8444**	**0.7988**	**0.8184**	**0.8957**
		BiLSTM	0.8816	0.8836	0.8825	0.8118	0.8002	0.8058	0.8836
**FastText**		CNN	0.8444	0.8524	0.8471	0.7633	0.7277	0.7426	0.8524
		BiLSTM	0.8521	0.8569	0.8538	0.7704	0.7467	0.7571	0.8569
**GloVe**	Task3	CNN	0.8593	0.8576	0.8538	0.7890	0.6981	0.7143	0.8576
		BiLSTM	**0.8445**	**0.8378**	**0.8373**	**0.7595**	**0.7201**	**0.7235**	**0.8378**
**FastText**		CNN	0.7758	0.7788	0.7717	0.6791	0.5813	0.6055	0.7788
		BiLSTM	0.7587	0.7600	0.7550	0.5937	0.5686	0.5664	0.7600

## Error analysis

In this section, we analyze the performance of the best-performing model for each subtask.

Table 11 presents the classwise scores for the best-performing models. For Subtask 1 and Subtask 2, the CNN with a Glove embedding. In Subtask 3, the best performance was achieved through soft-voting of models using only Unigram with TF-IDF values.

**Table 11:** Classwise scores for the best-performing models for each subtask, including sample sizes from Table 2.

Tasks	Model	Feature set	Label	Sample Size	Precision	Recall	F1-score
Subtask1	SVM (Linear)	All Features	Social Support	2236	0.7528	0.5750	0.6517
			Non-Social Support	7762	0.8854	0.9455	0.9144
Subtask2	CNN	GloVe	Group	1813	0.9198	0.9547	0.9369
			Individual	423	0.7691	0.6429	0.6999
			Black Community	114	0.8438	0.8684	0.8546
			LGBTQ	154	0.9199	0.8856	0.9012
Subtask3	Soft Voting	TF-IDF	Other Religion	520 19	0.7270 0.7333	0.7142 0.3452	0.7193 0.4233
			Women	24	0.7600	0.5214	0.5835
			Nation	982	0.8610	0.8913	0.8755

An additional column showing the sample sizes for each category (from Table 2) has been included in Table 11 to facilitate clearer comparison. The results indicate that although categories with larger sample sizes often achieve higher F1-scores, this trend is not consistent across all classes. For example, Nation (982 samples) obtained an F1-score of 0.8755, while LGBT (150 samples) achieved a higher F1-score of 0.9012. Similarly, the Black Community class, with a relatively small number of samples (114), reached an F1-score of 0.85. In contrast, categories such as Women and Religion showed both lower sample sizes and lower F1-scores. These observations indicate that while class imbalance has an impact on performance, certain minority classes can still be effectively recognized depending on their linguistic distinctiveness and internal consistency.

**Fig 2:**
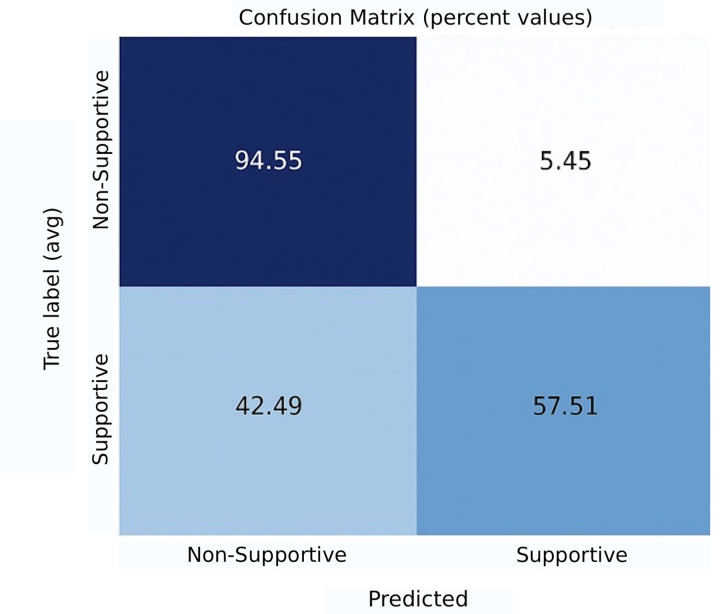
Confusion matrix for the best-performing model in subtask 1 (SVM (linear) + all features).

**Fig 3:**
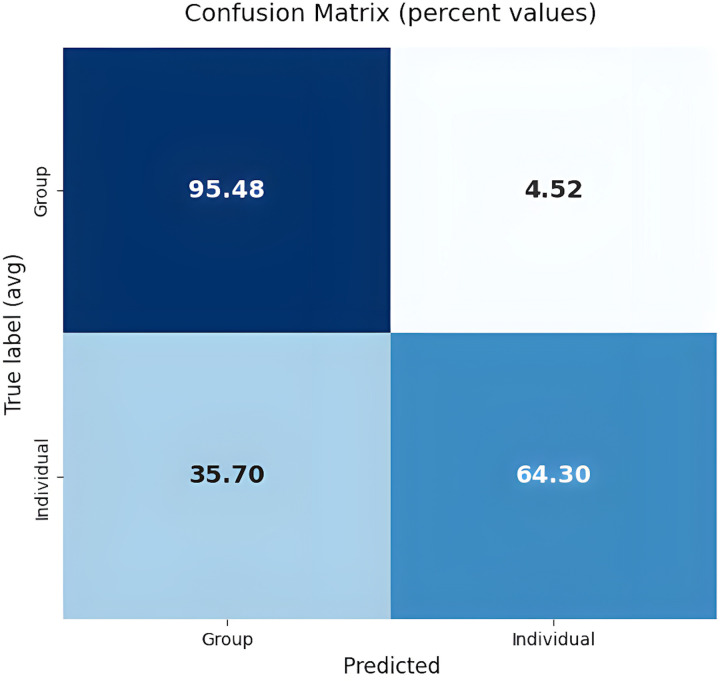
Confusion matrix for the best-performing model in subtask 2 (CNN + Glove).

**Fig 4:**
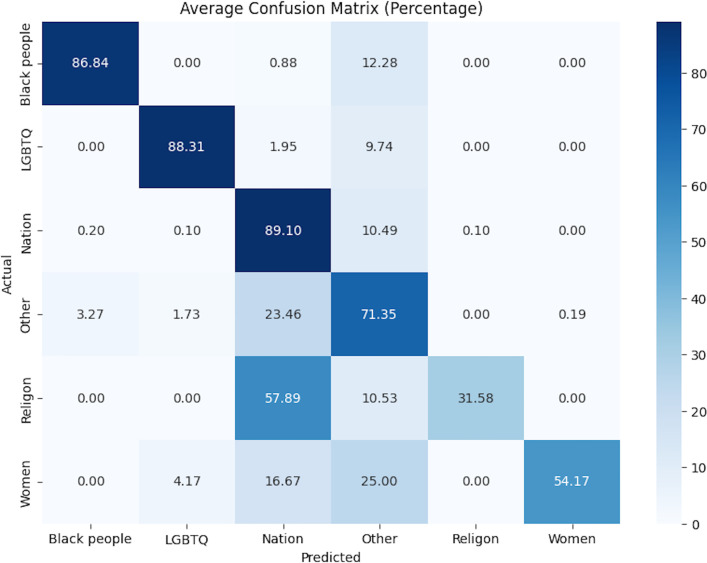
Confusion matrix for the best-performing model in subtask 3 (soft voting + TF-IDF).

We also provided the confusion matrices for these models in Figs 2–4. The analysis of these confusion matrices reveals several key patterns of misclassification.

For Subtask 1, supportive comments were frequently misclassified as non-supportive, whereas non-supportive comments were generally identified correctly. This asymmetry suggests that the model performs well at recognizing the absence of support but struggles to detect supportive expressions, likely due to their subtle linguistic or contextual cues.

In subtask 2, there was a notable confusion between support for individuals and support for groups. This suggests that the model finds it difficult to differentiate when the support is directed towards a single person versus a collective group, possibly because the expressions of support can be quite similar in both scenarios.

In subtask 3, when predicting targeted groups, the model often misclassified religious groups as nations. This specific confusion was more prominent than any other misclassification within this subtask. It indicates a particular challenge in distinguishing between religion-based and nation-based supportive comments, which may stem from overlapping cultural or contextual cues in the data.

Additionally, across all subtasks, there was significant misclassification into the”other” category. This category encompasses instances that may target multiple groups or do not fit neatly into predefined categories, making it a frequent source of confusion for the model.

## Discussion

The dataset and experiments proposed in this paper have several characteristics and limitations that are briefly discussed in the following:

- The dataset utilized in this paper was exclusively gathered from YouTube comments within a specific timeframe and targeted videos, which may introduce biases. To enhance the dataset’s diversity and richness, incorporating posts from other social media platforms, such as X and Reddit, in an open timeframe would be beneficial.
- The dataset is relatively small and imbalanced in terms of supportive comments, which has affected the performance of the machine learning models. In future work, this issue could be addressed by increasing the dataset size, particularly for supportive comments. We also observed that the class imbalance influenced model accuracy, as simple baselines using the majority class already achieved relatively high accuracy scores (e.g., 0.77 for Subtask 1 and 0.81 for Subtask 2). This highlights the importance of evaluating models beyond accuracy and focusing on metrics such as F1-score, especially in imbalanced settings.
- The current study reveals certain patterns in support expression within the analyzed dataset. However, since the videos and comments were not sampled to be representative of YouTube as a whole, we refrain from generalizing these observations to all YouTube content. Additionally, users show support for different nations without being heavily influenced by religious affiliations. The data indicates that recent support has not been predominantly directed towards any specific religion. Instead, people are more concerned with nations and communities, such as LGBTQ+ individuals and Black people. This trend highlights a broader social focus on national and community identities over religious considerations.
- This study serves as a foundational step in introducing the task of social support detection aimed at fostering support and positivity as an alternative to merely filtering out hate speech. Consequently, the paper primarily focuses on the introduction of this concept, assessing the feasibility of the task, and examining it from a psychological perspective. As such, experiments involving state-of-the-art models like transformers and large language models have been deferred to future works.
- It is important to note that the machine learning experiments for each subtask were conducted strictly on human-annotated subsets. For instance, only comments labeled as Social Support in Subtask 1 were used for further classification in Subtask 2, and likewise for Subtask 3. At no point were the predictions from previous subtasks used as inputs to subsequent ones. This design choice avoids the risk of cumulative error propagation across subtasks. While building a complete pipeline where predictions from one task feed into the next is a compelling direction for future research, such an experiment was not within the scope of the present study.

## Conclusion and future work

This study explores the dynamics of social support within online communities by introducing a structured classification framework and providing a labeled dataset to support further research. By introducing the task of SSD and conducting experiments on a dataset of YouTube comments, we identified patterns in supportive interactions, such as the relevance of psycholinguistic features and the effectiveness of combining linguistic and machine learning techniques for classification.

Our findings reveal that YouTube users predominantly express support for groups of people, with less emphasis on individual support and religious affiliations. This highlights the importance of considering broader societal contexts when analyzing social support interactions online.

While our experiments have provided promising results, there are notable limitations to address. The dataset used is relatively small and imbalanced, limiting the generalizability of our findings. Additionally, biases inherent in the dataset, stemming from its exclusive focus on YouTube comments within specific parameters, need to be addressed through the diversification of data sources.

Looking ahead, future research in SSD should focus on expanding and diversifying datasets by incorporating data from multiple platforms, such as Reddit and Twitter, to improve the dataset’s breadth and generalizability. Additionally, future work should explore advanced modeling techniques, such as n-grams, Transformer-based models, and large language models (LLMs), and also incorporate hyperparameter optimization for traditional machine learning models to enhance the performance and accuracy of SSD tasks. Another important direction for future work is investigating how insights from SSD can inform the design of interventions aimed at promoting positive interactions within online communities. Furthermore, we plan to address the current class imbalance by balancing the dataset using methods such as paraphrasing comments with GPT models. By carefully addressing these challenges and grounding future research in robust empirical evidence, we can deepen our understanding of social support dynamics online and contribute to fostering more supportive and inclusive digital spaces.

## Limitations

A key limitation of our study is the reliance on data collected from a single platform, YouTube, which may limit the broader applicability of our findings to other online environments. Additionally, the dataset exhibited class imbalance, with certain labels having a disproportionately low number of comments. This imbalance may have influenced the model’s performance, potentially skewing results towards the majority classes. Another limitation arises from our use of specific keywords to select 5,000 comments, which may have inadvertently excluded relevant comments containing different terms, thus limiting the diversity of the data.

Furthermore, the annotation process itself introduced challenges, particularly in distinguishing between individual and group support. For instance, a comment like “You are a true Italian!” may refer to personal (individual) support or to national identity (group) support—or both. This ambiguity posed difficulties for annotators. Our annotation guidelines instructed annotators to prioritize explicit cues: if the comment clearly referenced a social group (e.g., nationality, gender, religion), it was labeled as Group; otherwise, as Individual. In cases of uncertainty, we applied majority voting to determine the final label. These cases highlight the subjective nature of social support labeling and underscore the complexity of annotation in nuanced social contexts.

Finally, this study did not include spell-checking or correction during preprocessing.

Misspellings and orthographic variations common in YouTube comments may have affected the accuracy of lexical-based feature extraction. Future work could incorporate robust spell-correction methods to address this limitation.

## Supporting information

S1 Appendix YouTube videos and comment date ranges. Description: This appendix provides the list of YouTube videos used and their corresponding comment date ranges.
(DOCX)

S2 Appendix Keywords used for supportive content sampling. Description: This appendix contains the list of keywords and phrases used to identify potentially supportive comments.
(DOCX)
