# Supplementary material for: Social support detection from social media texts
Source: PLoS One. 2026 Mar 25;21(3):e0337476. doi: 10.1371/journal.pone.0337476 (PMC13016356; doi:10.1371/journal.pone.0337476)
Supplement: S1 Appendix — Description: This appendix provides the list of YouTube videos used and their corresponding comment date ranges. (DOCX) [file pone.0337476.s001.docx]

# Appendix A: YouTube Videos and Comment Dates

| # | Video URL | Dates |
| --- | --- | --- |
| 1 | https://www.youtube.com/watch?v=j2cvighfeBE | Jun 15, 2020 – Feb 13, 2024 |
| 2 | https://www.youtube.com/watch?v=KOdzMd1KLrg | Jul 24, 2018 – Feb 13, 2024 |
| 3 | https://www.youtube.com/watch?v=-YkU_NcV0NE | Jan 4, 2020 – Feb 13, 2024 |
| 4 | https://www.youtube.com/watch?v=Ak4GR9qGi-Y | Sep 30, 2022 – Feb 13, 2024 |
| 5 | https://www.youtube.com/watch?v=FTHClTOA5MU | Jan 27, 2024 – Feb 13, 2024 |
| 6 | https://www.youtube.com/watch?v=1aRFQ30ME18 | Jan 29, 2024 – Feb 13, 2024 |
| 7 | https://www.youtube.com/shorts/AY-ODE82OeY | Oct 28, 2023 – Feb 13, 2024 |
| 8 | https://www.youtube.com/watch?v=aG80TQ_-Ffs | Mar 5, 2023 – Feb 13, 2024 |
| 9 | https://www.youtube.com/watch?v=NH8nhqQbd28 | May 27, 2020 – Feb 13, 2024 |
| 10 | https://www.youtube.com/watch?v=mI7eHX9u4Q0 | Apr 12, 2016 – Feb 13, 2024 |
| 11 | https://www.youtube.com/watch?v=IuASG9C7Jb4 | Jul 13, 2016 – Feb 13, 2024 |
| 12 | https://youtube.com/shorts/t7WZI2x5M_s?si=Zy5Mh5vRnp7A-ZJ2 | Feb 22, 2023 – Feb 13, 2024 |
| 13 | https://www.youtube.com/watch?v=aG80TQ_-Ffs&t=442s | Apr 3, 2023 – Feb 13, 2024 |
| 14 | https://www.youtube.com/watch?v=meOWV1w2GE8&t=521s | Jul 16, 2023 – Feb 13, 2024 |
| 15 | https://www.youtube.com/watch?v=anD9ZrMJT8k | Apr 9, 2018 – Feb 13, 2024 |
