# Supplementary material for: Social support detection from social media texts
Source: PLoS One. 2026 Mar 25;21(3):e0337476. doi: 10.1371/journal.pone.0337476 (PMC13016356; doi:10.1371/journal.pone.0337476)
Supplement: S2 Appendix — Description: This appendix contains the list of keywords and phrases used to identify potentially supportive comments. (DOCX) [file pone.0337476.s002.docx]

Appendix B: Keywords Used for Supportive Content Sampling

The following keywords and phrases were used to identify potentially supportive comments

in our sampling process. These terms were selected based on their semantic relevance to

the concept of social support and were informed by both prior literature and manual inspection.

• you can do it

• stay strong

• i understand

• you’re not alone

• sending positive vibes

• i’m here to help

• keep your head up

• things will get better

• i believe in you

• support

• inspiring

• stand

• encouragement

• comfort

• empathy

• positivity

• assistance

• help

• aid

• understanding

• compassion

• kindness

• sympathy

• hope
